# Adaptation of the International Fitness Scale and Self-Perceived Health-Related Physical Fitness Questionnaire into Turkish

**DOI:** 10.3390/children10091546

**Published:** 2023-09-13

**Authors:** Tuba İnce Parpucu, Görkem Kıyak, Fatih Uğur Taş, Mert Usta, Aydan Örsçelik, Sabriye Ercan

**Affiliations:** 1 Department of Physiotherapy and Rehabilitation, Faculty of Health Sciences, Süleyman Demirel University, Isparta 32260, Türkiye; fzttubaince@gmail.com (T.İ.P.); mertusta@sdu.edu.tr (M.U.); 2 Department of Sports Medicine, Faculty of Medicine, Süleyman Demirel University, Isparta 32260, Türkiye; gorkemkiyak@sdu.edu.tr (G.K.); fatihtas@sdu.edu.tr (F.U.T.); 3 Department of Sports Medicine, Gülhane Medical Faculty, Health Sciences University, Ankara 06010, Türkiye; draydanorscelik@gmail.com

**Keywords:** physical fitness, validity, reliability, adolescent

## Abstract

This study aims to investigate the Turkish validity and reliability of the International Fitness Scale (IFIS) and the Self-Perceived Health-Related Physical Fitness Questionnaire for Children (SPHQ-C) aged 10–17. The measurement tools were translated into Turkish by two independent researchers and back-translated. Then, the content validity was established with a group of 13 individuals (Sample 1) who presented to the sports medicine clinic. The pilot application phase was initiated. A sample of 372 individuals (Sample 2) studying in the provincial center was selected for piloting the measurement tools with the ALPHA-FIT Test Battery. The test–retest application of the measurement tools with a 15-day interval was carried out with 207 individuals (Sample 3) not included in Sample 2. The authors calculated that the content validity ratio values for the items in the IFIS ranged from 0.85 to 1.00, with a content validity index of 0.94 for the scale. In the SPHQ-C, these values were found to be between 0.92 and 1.00, with a content validity index of 0.99. According to the Z-score difference analysis for both measurement tools, the construct validity of all items was achieved (*p* < 0.05). In the Bland–Altman plot test conducted for the IFIS-TR, the mean difference was calculated as 0.11, the standard deviation of the differences was 1.57, and the *p*-value was determined as 0.31. For the SPHQ-C-TR, the mean difference was −0.15, the standard deviation of the differences was 1.95, and the *p*-value was determined as 0.26. The Kappa coefficients for the items of the IFIS-TR ranged from 0.45 to 0.52, while for the SPHQ-C-TR, the Kappa coefficients ranged from 0.47 to 0.75. Confirmatory factor analysis conducted on Sample 2 confirmed model fit indices with covariance among some items of the measurement tools (IFIS-TR: e1–e3; SPHQ-C-TR: e1–e2, e1–e3, e2–e3, e3–e4, e6–e7, e8–e9). The IFIS and SPHQ-C measurement tools have been adapted into Turkish, and their validity and reliability have been established.

## 1. Introduction

Physical fitness is a concept that encompasses an individual’s health and fitness status while performing daily activities [1]. According to the American College of Sports Medicine, physical fitness is defined as the ability to perform desired physical activities according to the intended purpose [1]. This concept is influenced by a range of components such as body composition, flexibility, muscle strength, agility, speed, and endurance [1]. Adequate levels of parameters such as cardiovascular fitness, muscular strength, power, agility, coordination, and flexibility are necessary to fulfill the required physical performance during activities [2,3,4].

Physical fitness is important for both assessing the status compared to peers and predicting potential problems in adulthood for children and adolescents. In this context, the level of physical fitness in children and adolescents is an important component and determinant of health [4,5,6,7]. Thereby, there is a need to measure the physical fitness status of individuals or groups for various purposes such as medical screenings, injury risk analysis, muscle strengthening, rehabilitation programs and education interventions [8]. Different tests have been defined to be performed under field/laboratory conditions or with/without equipment. Among them, field tests performed without equipment stand out due to their practical advantages [8,9]. Several test batteries suitable for field use have been developed to assess physical fitness. Some of them include the functional fitness test series prepared by the American Alliance for Health, Physical Education, and Recreation (AAHPER) [10], the health-related physical fitness test series developed by the Urho Kaleva Kekkonen (UKK) Institute [8], and the ALPHA-FIT Test Battery [9].

Although these test batteries are measurement tools with established validity and reliability, there may be challenges in their application in the field, such as lack of time, space, and availability of professional testers. Some of these challenges include the time-consuming nature of the testing process and the unavailability of suitable locations with the required characteristics due to various geographical, social, and economic reasons. On the other hand, the results of performance tests may be affected by the internal and external motivational factors of the athlete being tested, as well as the gender of the tester, and somatic characteristics may be affected [11]. In addition, although it is thought that the physical fitness levels of healthy individuals and the performance test results will be parallel, individuals who are underweight can also perform better than those with overweight/obesity, similar to individuals with a normal body weight [12]. Therefore, there is a need for scales that can inform individuals’ physical fitness [13]. Measurement tools that assess individuals’ self-perceived fitness and health status allow for evaluations that can inform the physical fitness of children and adolescent age groups without the need for field tests. The International Fitness Scale (IFIS) [14] and the Self-Perceived Health-Related Physical Fitness Questionnaire for Children (SPHQ-C) [15] are measurement tools developed for this purpose. These measurement tools have shown that information about the physical fitness levels of school-age children can be obtained without the need for field tests [14,15].

As far as we know, there is no valid and reliable measurement tool for children and adolescent in Turkish that can inform the level of physical fitness by adapting to field tests. This study aims to establish the Turkish validity and reliability of the IFIS and the SPHQ-C in children aged 10–17.

## 2. Materials and Methods

Permission was obtained from the developers of the IFIS [14] and the Self-Perceived Health-Related Physical Fitness Survey [15] via email for this research to take place. Following approval from the local ethics committee (dated 27 October 2022, number 305), permission was obtained from the Governor’s Office (dated 25 November 2022, number 64255721). All individuals participating in the study did so voluntarily and provided consent, along with consent from their families, to participate in the research.

### 2.1. Study Population

After the translation of the measurement tools from their original languages to Turkish by two independent researchers, and the completion of the back-translation process, the comprehensibility of the measurement tools was evaluated during the content validity phase. The study’s population for evaluating content validity consisted of 13 individuals (Sample 1) who presented to the sports medicine outpatient clinic. The pilot application phase was initiated after ensuring content validity for both measurement tools.

For the pilot application of the measurement tools and the ALPHA-FIT Test Battery [9], 372 individuals (*n* = 188, 50.5% female; *n* = 184, 49.5% male) with an average age of 13.24 ± 0.90 years, who received education in the city center, were selected (Sample 2).

The test–retest application of the measurement tools, with a 15-day interval, was conducted with 207 individuals (*n* = 110, 53.1% female; *n* = 97, 46.9% male) who were not included in Sample 2, received education in the city center, and had an average age of 12.56 ± 0.12 years (Sample 3) (Table 1).

In the construct/predictive validity and reliability research of the tool, it is considered sufficient to reach a sampling of 5–20 times the number of items in the scale/questionnaire for the strength of the sample [16]. According to this information, in our study, we aimed to reach a sample covering more than 20 times the number of items on the tools, and it was accepted that it provided the level of competence for the sample size.

### 2.2. Data Collection Instruments and Methods

The individuals in Sample 2 who voluntarily participated in the study and had no illness that would hinder participation in sports filled out an information form containing descriptive characteristics and measurement tools that underwent Turkish validity and reliability studies through face-to-face interviews. After completing the measurement tools, individuals whose resting blood pressure (Erka, Perfect Aneroid, Bad Tölz, Germany) was measured twice were included in the physical fitness tests within the ALPHA-FIT Test Battery. During this stage, calculations were made for mean arterial pressure (the average arterial pressure = diastolic blood pressure + [0.333 × (systolic blood pressure − diastolic blood pressure)]), body composition measurements (Seca 700 Sliding Weight Mechanical Scale, Hamburg, Germany; Baseline 12-1110 Medical Skinfold Caliper, New York, NY, USA; Sevinç Mezura 272, İstanbul, Türkiye), sit-and-reach test, hand grip strength measurement (Baseline 300 lbs Hydraulic Hand Dynamometer, New York, NY, USA), standing long jump test, a 4 × 10 m shuttle run test, and a 20 m shuttle run test [9].

The individuals in Sample 3 completed the information form containing descriptive characteristics and the measurement tools that underwent Turkish validity and reliability studies through face-to-face interviews on the first day (0th test day) and then responded to the same questions again on the 15th day (retest day).

### 2.3. Statistical Analysis

The content validity of the measurement tools was examined using the Davis method [16]. The content validity ratio and the content validity index values specific to each item were calculated. A critical reference value of 0.80 or higher was used as the criterion for decision making [16].

The construct validity of the measurement tools, as demonstrated by the ALPHA-FIT Test Battery, was tested using a one-way ANOVA [17]. The prediction and reliability of the measurement tools were tested using the data obtained during the test–retest phase [16]. The Bland–Altman plot test and Cohen’s Kappa coefficient were used for this purpose [18]. The model fit of the measurement tools to their originals was determined through confirmatory factor analysis [19,20]. SPSS v.23 and AMOS v.24 software programs were used for the analyses. The data were presented as frequencies, percentages, and means with standard errors. A *p*-value of 0.05 was considered statistically significant [21].

## 3. Results

### 3.1. Content Validity

The content validity of the measurement tools was examined with 13 individuals aged 14 ± 0.59 years (*n* = 7, 53.8% female; *n* = 6, 46.2% male). In Sample 1, 30.8% (*n* = 4) of these individuals reported not engaging in regular exercise, while 69.2% (*n* = 9) stated that they had been exercising for 1.77 ± 0.42 years, spending an average of 295.38 ± 66.38 min per week.

Thus, the content validity values of the items in the IFIS ranged from 0.85 to 1.00, with a content validity index of 0.94 calculated for the scale. In the SPHQ-C, these values were found to range from 0.92 to 1.00, with a content validity index of 0.99.

### 3.2. Construct Validity

The data from Sample 2 were used to analyze construct validity for this study (Table 2). Among these individuals (*n* = 372), 56.5% (*n* = 210) reported not engaging in regular exercise, while 43.5% (*n* = 162) reported exercising for 1.66 ± 0.14 years, spending an average of 160.49 ± 14.19 min per week.

The response to each item was grouped according to the self-reported fitness level in the measurement instruments, and Z-score difference analysis was conducted on the physical fitness results. Significant differences were observed in the context of the IFIS-TR item 1, waist circumference (*p* = 0.008), and body fat percentage (*p* < 0.001) among the five categorized groups (very poor, poor, average, good, very good) based on self-reported fitness responses. Similarly, for the IFIS-TR item 2, the result of the 20 m shuttle run test (*p* < 0.001) showed significant differences. The IFIS-TR item 3 exhibited differences in hand grip strength (*p* < 0.001) and standing long jump distance (*p* < 0.001) based on self-reported fitness responses. On the other hand, the IFIS-TR item 4 and item 5 were analyzed within the four categorized groups (poor, average, good, very good) based on self-reported fitness responses. Significant differences were found in the IFIS-TR item 4 with the result of the 4 × 10 m shuttle run test (*p* < 0.001), and the IFIS-TR item 5 showed differences in the sit and reach test result (*p* = 0.001).

Similar results were observed in the anticipated physical fitness test results for the SPHQ-C-TR items. Significant differences were found in the grouped results based on self-reported fitness responses in the five categorized groups (very poor, poor, average, good, very good) for the SPHQ-C-TR item 1 with hand grip strength (*p* < 0.001), item 2 with the 20 m shuttle run test (*p* = 0.002), item 3 with standing long jump distance (*p* < 0.001), item 4 with the 20 m shuttle run test (*p* < 0.001) and 4 × 10 m shuttle run test (*p* < 0.001), item 5 with the 20 m shuttle run test (*p* < 0.001), item 6 with the sit and reach test (*p* < 0.001), and item 8 with body mass index (*p* < 0.001), waist circumference (*p* < 0.001), and body fat percentage (*p* < 0.001). In the four categorized groups (very poor, poor, average, good) based on self-reported fitness responses, significant differences were observed for the SPHQ-C-TR item 7 with the sit and reach test (*p* < 0.001), and item 9 showed differences in body mass index (*p* < 0.001), waist circumference (*p* < 0.001), and body fat percentage (*p* < 0.001) values.

### 3.3. Predictive Validity

In the stage of predictive validity and reliability analysis, data from Sample 3 were utilized. Among the individuals in Sample 3 (*n* = 207), 53.1% (*n* = 110) reported no participation in exercise, while 46.9% (*n* = 97) declared engaging in exercise for 2.86 ± 0.22 years, with a weekly duration of 135.89 ± 16.25 min.

**Figure 1:**
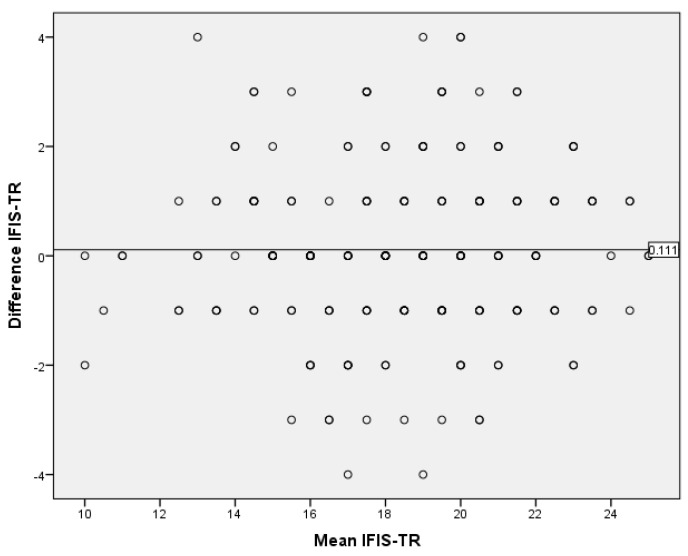
Bland–Altman plot graph of the International Fitness Scale—TR.

For the IFIS-TR, a Bland–Altman plot test was conducted, yielding a mean difference of 0.11 (95% confidence interval, lower bound: −0.11; upper bound: 0.33) and a standard deviation of 1.57, with a *p*-value of 0.31. Consequently, the scale’s results were found to exhibit a stable distribution within the range of −2.97 to +3.19, with a 95% confidence interval (Figure 1).

**Figure 2:**
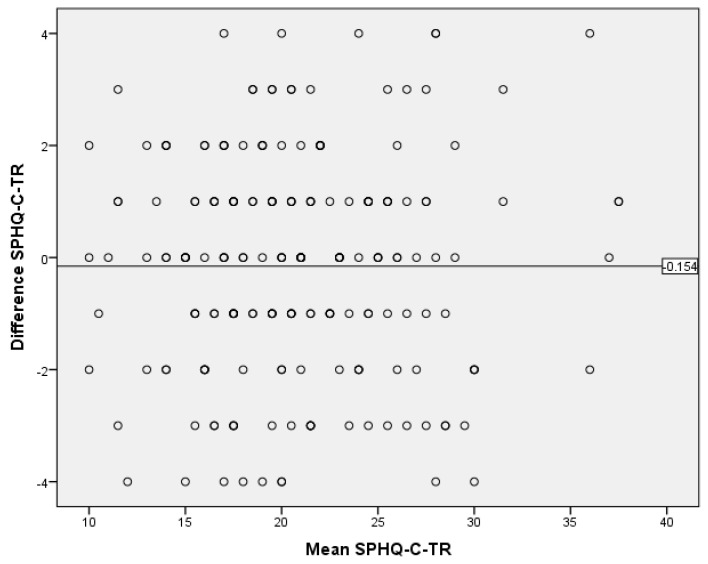
Bland–Altman plot graph of the Self-Perceived HRPF Questionnaire for Children—TR.

According to the Bland–Altman plot test results for the SPHQ-C-TR, the mean difference was −0.15 (95% confidence interval, lower bound: −0.42; upper bound: 0.11), with a standard deviation of 1.95 and a *p*-value of 0.26. Thus, the questionnaire’s results demonstrated stability with a distribution within the range of −3.97 to +3.67, with a 95% confidence interval (Figure 2).

### 3.4. Reliability

The Cohen’s Kappa coefficients were assessed for each item of the measurement instruments. As a result, the items of the IFIS-TR exhibited Kappa coefficients in the range of 0.45–0.52. In the SPHQ-C-TR, the Kappa coefficients ranged from 0.47 to 0.75. Thus, it was determined that the items demonstrated a good level of agreement [16] (Table 3).

### 3.5. Confirmatory Factor Analysis

Confirmatory factor analysis was conducted using the responses from Sample 2 as a reference. Covariances were established among certain items (IFIS-TR: e1–e3; SPHQ-C-TR: e1–e2, e1–e3, e2–e3, e3–e4, e6–e7, e8–e9) to verify the measurement instruments. Thus, it was observed that the theoretical structure in the original measurement instruments was preserved in the Turkish versions of the measurement instruments, as indicated by the goodness-of-fit indices presented in Table 4.

## 4. Discussion 

This study aimed to investigate the Turkish validity and reliability of the IFIS and the SPHQ-C in children aged 10–17. The content validity ratio values for the items in IFIS ranged from 0.85 to 1.00, with a content validity index of 0.94 for the scale. In the SPHQ-C, these values were found to be between 0.92 and 1.00, with a content validity index of 0.99. According to the Z-score difference analysis for both measurement tools, the construct validity of all items was achieved (*p* < 0.05). In the Bland–Altman plot test for the IFIS and SPHQ-C, the consistency of the measurements was demonstrated. The Kappa coefficients for the items of the IFIS-TR ranged from 0.45 to 0.52, while for the SPHQ-C-TR, the Kappa coefficients ranged from 0.47 to 0.75. Confirmatory factor analysis conducted on Sample 2 confirmed model fit indices. This study establishes the Turkish validity and reliability of both measurement instruments. By comparing the measurement instruments with the ALPHA-FIT Test Battery results, which are field-based measurements devoid of sociocultural and linguistic differences, it was observed that the theoretical structure in the original measurement instruments was well-preserved in their Turkish versions. Consequently, when field-based measurements of physical fitness parameters cannot be conducted, the use of self-administered measurement instruments by children/adolescents can facilitate the understanding of their overall physical condition in a short period of time.

### 4.1. Content Validity

The first step in testing the validity of measurement instruments is evaluating comprehensibility, which falls under content validity. Various methods have been defined to determine comprehensibility, with the Davis method commonly used, where the critical value is set at 0.80 [16]. The results of the pilot study for content validity showed that the calculated values for both the IFIS and the SPHQ-C exceeded the critical threshold. Based on these data, it was observed that both measurement instruments adequately encompass the concept of physical fitness they aim to measure and are well understood by Turkish children/adolescents.

### 4.2. Construct Validity

To ensure construct validity of the measurement instruments, the ALPHA-FIT Test Battery [9] was employed. The current study with Turkish children and adolescents demonstrated statistically significant differences in all items of both measurement instruments compared to the relevant ALPHA-FIT Test Battery measurements. These findings are consistent with the previous literature reporting good construct validity for both measurement instruments [14,15,22,23]. As a result, it is understood that both measurement instruments can be used to inform the physical fitness parameters of these age groups.

### 4.3. Predictive Validity

In this study, the predictive validity of both measurement instruments was demonstrated by analyzing the responses given to the measurement instruments administered at a two-week interval. The measurement instruments showed temporal stability within an approximate range of ±4 points. It has been determined in the literature that the temporal stability of the measurement tools in question has not been evaluated with the Bland–Altman plot test. However, if the lower and upper bounds determined according to the Bland–Altman plot test are at a clinically acceptable level, the new method can be used in daily practice [24]. Thereby, the determined level of deviation is considered valuable for informing the physical fitness levels of children/adolescents when field tests cannot be conducted. The observed deviation in scores due to the adaptation of measurement instruments to different cultures should be discussed in line with the literature in future research.

### 4.4. Reliability

The items of the IFIS-TR formed Kappa coefficients in the range of 0.45–0.52, while the items of the SPHQ-C-TR formed Kappa coefficients in the range of 0.47–0.75, indicating good reliability of both measurement instruments in Turkish [16]. Similarly, in the original IFIS study [14], Kappa coefficients were reported as 0.54–0.65, and in the original SPHQ-C study [15], Kappa coefficients were reported as 0.53–0.76. Considering these data, it was determined that both measurement instruments are reliable in their Turkish versions, just as they are in their original versions. These findings support that the measurement tools can be used safely in Turkish culture as well as in their originals.

### 4.5. Confirmatory Factor Analysis

When adapting original measurement tools to other cultures, confirmatory factor analysis is performed as part of the research methodology to verify [16]. By establishing covariances among items that showed covariation with each other, the structure of both measurement instruments for Turkish children and adolescents was validated. It was observed that the theoretical structure developed in the original measurement instruments was preserved in their Turkish versions. This finding is the last methodological step in validity and reliability research, and the compatibility of this stage with the original tools will support the widespread use of Turkish versions in practice.

### 4.6. Limitations

One limitation of our current study is the inability to obtain a sample encompassing all of Turkey’s geographical regions. This may have created a lack of diversity in the sample. It would be beneficial to replicate this research by reaching larger populations of Turkish children and adolescents, who are spread across a vast geography and have diverse socio-demographic backgrounds. In future studies, the evaluation of measurement tools in areas with different socio-demographic characteristics can help support our findings.

## 5. Conclusions

As a result of this research, the IFIS (Supplementary Materials S1) and SPHQ-C (Supplementary Materials S2) measurement tools have been adapted into Turkish, and their validity and reliability have been established. We recommend using these measurement tools to inform the physical fitness levels of children and adolescents in situations where field tests such as the ALPHA-FIT Test Battery cannot be conducted, or objective measurement methods are hindered by material and/or environmental constraints.

## Figures and Tables

**Table 1:** Descriptives of samples.

	Sample 1	Sample 2	Sample 3
Process	Content validity	Construct validity	Predictive validity and reliability
Sample size, *n*	13	372	207
Age, years	14 ± 0.59	13.24 ± 0.90	12.56 ± 0.12
Gender, *n* (%)			
Female	7, 53.8%	188 (50.5%)	110, 53.1%
Male	6, 46.2%	184 (49.5%)	97, 46.9%
Regular exercise, *n* (%)			
No	4, 30.8%	210, 56.5%	110, 53.1%
Yes/years	9, 69.2%/1.77 ± 0.42	162, 43.5%/1.66 ± 0.14	97, 46.9%/2.86 ± 0.22

**Table 2:** Results of the physical fitness levels in Sample 2.

	Mean (SE)
Average arterial pressure (mmHg)	88.82 ± 0.48
Body mass index (kg/m^2^)	19.79 ± 0.21
Waist circumference (cm)	68.18 ± 0.51
Body fat percentage (%)	21.25 ± 0.39
Sit and reach test (cm)	18.76 ± 0.36
Hand grip strength (kg)	22.83 ± 0.39
Standing long jump distance (cm)	112.01 ± 1.49
4 × 10 m shuttle run test (s)	14.17 ± 0.08
20 m shuttle run test (mL/min/kg)	28.34 ± 0.24

SE: standard error.

**Table 3:** Reliability of the International Fitness Scale—TR and the Self-Perceived HRPF Questionnaire for Children—TR.

IFIS-TR	Test	Retest	Kappa Coefficients (SE)	SPHQ-C-TR	Test	Retest	Kappa Coefficients (SE)
Item 1	3.75 ± 0.06	3.71 ± 0.06	0.45 ± 0.05	Item 1	2.42 ± 0.06	2.35 ± 0.06	0.49 ± 0.05
Item 2	3.74 ± 0.07	3.71 ± 0.07	0.47 ± 0.05	Item 2	2.23 ± 0.05	2.32 ± 0.06	0.47 ± 0.05
Item 3	3.56 ± 0.06	3.64 ± 0.06	0.52 ± 0.05	Item 3	2.55 ± 0.05	2.57 ± 0.05	0.65 ± 0.05
Item 4	3.89 ± 0.06	3.96 ± 0.06	0.47 ± 0.05	Item 4	2.20 ± 0.09	2.11 ± 0.08	0.48 ± 0.05
Item 5	3.36 ± 0.07	3.39 ± 0.07	0.47 ± 0.05	Item 5	2.51 ± 0.06	2.46 ± 0.06	0.47 ± 0.05
				Item 6	2.35 ± 0.07	2.39 ± 0.08	0.49 ± 0.05
				Item 7	2.84 ± 0.06	2.77 ± 0.06	0.56 ± 0.05
				Item 8	2.87 ± 0.06	2.88 ± 0.06	0.75 ± 0.04
				Item 9	3.08 ± 0.05	3.06 ± 0.05	0.73 ± 0.05

SE: standard error.

**Table 4:** Goodness-of-fit indices for the IFIS-TR and the SPHQ-C-TR.

	Value		Diagram	
Model Fit Indices	IFIS-TR	SPHQ-C-TR	IFIS-TR	SPHQ-C-TR
**χ^2^**	5.95	77.38	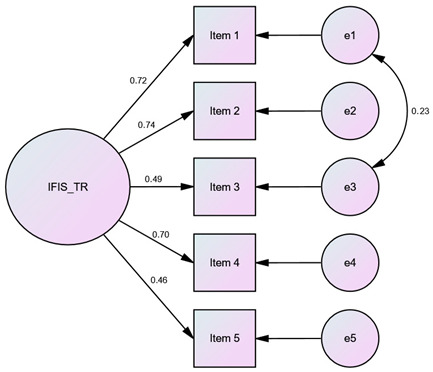	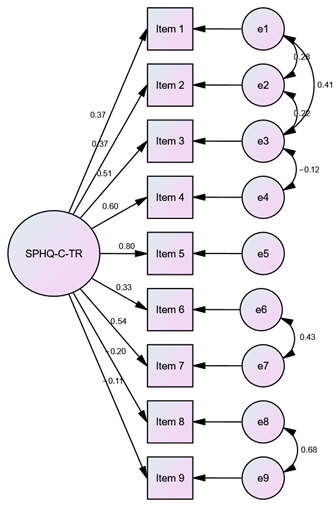
**df**	4	21		
**p**	0.20	˂0.001		
**χ^2^/df**	1.49	3.69		
**SRMR**	0.02	0.05		
**RMSEA**	0.04	0.09		
**GFI**	0.99	0.96		
**AGFI**	0.98	0.91		
**CFI**	0.99	0.93		
**NFI**	0.99	0.91		
**TLI**	0.99	0.89		
**IFI**	0.99	0.94		

**Abbreviation:** df = degree of freedom; SRMR = standardized root means square residual; RMSEA = root mean square error of approximation; GFI = goodness-of-fit index; AGFI = adjusted goodness of fit index; CFI = comparative fit index; NFI = normed fit index; TLI = Tucker–Lewis index; IFI = incremental fit index.

## Data Availability

The dataset analyzed in this study can be requested from corresponding author on reasonable request.

## Supplementary Materials

The following supporting information can be downloaded at: https://www.mdpi.com/article/10.3390/children10091546/s1.
