# Supplementary material for: Adaptation of the International Fitness Scale and Self-Perceived Health-Related Physical Fitness Questionnaire into Turkish
Source: Children (Basel). 2023 Sep 13;10(9):1546. doi: 10.3390/children10091546 (PMC10528336; doi:10.3390/children10091546)
Supplement: Supplementary file 1 [file children-10-01546-s001.zip › children-2601411-supplementary.pdf]

children

MDPI

Supplementary Materials

Supplementary Materials S1. Turkish Version of International Fitness Scale

Uluslararası Fiziksel Uygunluk Ölçeği (UFUÖ).

KENDİ KENDİNE ALGILANAN FİZİKSEL UYGUNLUK

Sınıf arkadaşlarınızın verdiği cevapları dikkate almadan bu testi tek başınıza yapmanız çok önemlidir. Cevabınız sadece bilimin ve tıbbın ilerlemesi için kullanılacaktır. Lütfen tüm soruları cevaplayınız ve boş bırakmayınız. Her soru için yalnızca bir seçeneği işaretleyiniz ve daha da önemlisi (soruları cevaplarken) samimi olunuz. İş birliğiniz için teşekkür ederiz.

Lütfen fiziksel uygunluk seviyenizi (arkadaşlarınıza kıyasla) düşünmeye çalışınız ve doğru seçeneği seçiniz.

Genel fiziksel uygunluğunuz:
Çok kötü
Kötü
Orta
İyi
Çok iyi

Dolaşım ve solunum sistemi uygunluğunuz (örneğin uzun süre koşma gibi egzersiz yapma kapasiteniz):
Çok kötü
Kötü
Orta
İyi
Çok iyi

Kas kuvvetiniz:
Çok kötü
Kötü
Orta
İyi
Çok iyi

Hızınız/çevikliğiniz:
Çok kötü
Kötü
Orta
İyi
Çok iyi

Esnekliğiniz:
Çok kötü
Kötü
Orta
İyi
Çok iyi

ZAMAN AYIRDIĞINIZ VE KATILIMINIZ İÇİN TEŞEKKÜR EDERİZ

Supplementary Materials S2. Turkish Version of Self-Perceived Health-Related Physical Fitness Questionnaire

Çocuklar İçin Kendi Kendine Algılanan Sağlıkla İlgili Fiziksel Uygunluk Anketi (ASFU-Ç).

Sınıf arkadaşlarınızın verdiği cevapları dikkate almadan bu testi tek başınıza yapmanız çok önemlidir. Cevabınız sadece bilimin ve tıbbın ilerlemesi için kullanılacaktır. Lütfen tüm soruları cevaplayınız ve boş bırakmayınız. Her soru için yalnızca bir seçeneği işaretleyiniz ve daha da önemlisi (soruları cevaplarken) samimi olunuz. İş birliğiniz için teşekkür ederiz.

Lütfen fiziksel uygunluk seviyenizi düşünmeye çalışınız ve doğru seçeneği seçiniz.

	**Çok ağır bir şeyi yerden kaldırmam istenirse (büyük bir taş, 6 kutu süt içeren bir kasa, 6 litrelik su şişesi vb.)**
1	Sorunsuz yaparım ve düşürmeden uzun süre tutabilirim, çünkü çok güçlüyüm.
2	Sorunsuz yaparım ve düşürmeden oldukça uzun süre tutabilirim, çünkü oldukça güçlüyüm.
3	Güçlükle yapabilsem de düşürmeden tutabilirim, çünkü gücüm normal seviyededir.
4	Yaparım ama hemen yere düşerler, çünkü gücüm azdır.
5	Çok ağır yükleri yerden kaldıramam, çünkü gücüm çok azdır.
	**Elimde kitap dolu iki sırt çantasıyla okul gibi bir yerde merdivenlerden ikinci kata çıkmak zorunda kalsaydım (her elde bir çanta olacak şekilde)**
1	Dinlenmek için durmadan ve yardım almadan tek başıma çıkarım çünkü çok güçlüyüm.
2	Dinlenmek için durmak zorunda kalmadan merdivenlerden tek başıma çıkarım çünkü oldukça güçlüyüm ama biraz yorulurum.
3	Bazen dinlenmek için durmak zorunda kalsam da tek başıma merdivenlerden çıkabilirim çünkü gücüm normal seviyededir.
4	Onları birinci kata kadar çıkarırım, sonra bana yardım etmek zorunda kalırlardı, çünkü gücüm azdır.
5	Çantaları taşıması için birisi bana yardım etmek zorunda kalırdı, çünkü hiç gücüm yok.
	**Benim yaşımdaki diğer erkeklerle/kızlarla kıyaslandığımda…**
1	En güçlü olan benim.
2	En güçlü olanlardan biriyim.
3	Ne en güçlüyüm ne de en az güçlüyüm, gücüm normal seviyededir.
4	En az güce sahip olanlardan biriyim.
5	En az güce sahip olan benim.
	**Beden eğitimi dersinde hafif bir tempoda koşmak zorunda kalırsam…**
1	Ders boyunca, hiç durmadan ve yorulmadan koşabilirim.
2	Yorulmamak için biraz ara vermem gerekse de ders boyunca koşabilirim.
3	Biraz ara vererek ders süresinin neredeyse yarısı boyunca koşabilirim.
4	Birkaç kez dinlenmek için ara vererek ders süresinin yaklaşık yarısı boyunca koşabilirim.
5	Ara sıra durarak 5–10 dakika koşabilirim. Sonrasında çok fazla koşamayacağım için devam edemem.
	**Benim yaşımdaki diğer erkeklerle/kızlarla kıyaslandığımda…**
1	Dinlenmek için durmak zorunda kalmadan en uzun süre egzersiz yapan kişi benim.
2	Dinlenmek için durmak zorunda kalmadan en uzun süre egzersiz yapabilen kişilerden biriyim.
3	Dinlenmek için durmak zorunda kalmadan ne en uzun süre ne de en az süre egzersiz yapabilen kişilerden biriyim.
4	Dinlenmek için durmak zorunda kalmadan en az süre egzersiz yapabilen kişilerden biriyim.
5	Dinlenmek için durmak zorunda kalmadan en az egzersiz yapan kişi benim.
	**Bacaklarım düz ve birleşik pozisyonda iken öne doğru uzanarak ayaklarımın ucuna dokunmaya çalıştığımda…**

1	Sorunsuz bir şekilde yapabiliyorum ve çok esnek olduğum için ayak parmaklarımın ucunu geçerek ayak tabanlarıma bile dokunabiliyorum.
2	Ayak parmaklarımın ucunu geçerek ayak tabanlarıma dokunmak benim için zor olsa da oldukça esnek olduğum için sorunsuz yapabilirim.
3	Bunu yapmak benim için zor, çünkü denediğimde bacak kaslarım biraz "çekiyor".
4	Bunu zor yapıyorum (neredeyse yapamıyorum) çünkü denediğimde bacak kaslarım çok fazla "çekiyor" ve ayrıca az esnekliğim var.
5	Ne kadar denersem deneyeyim bunu yapamam, çünkü yaptığımda bacak kaslarım çok fazla "çekiyor" ve ayrıca çok az esnekliğim var.
	**Benim yaşımdaki diğer erkeklerle/kızlarla kıyaslandığımda…**
1	En esnek olan benim.
2	En esnek olanlardan biriyim.
3	Ne en fazla esnekliğe ne de en az esnekliğe sahip olanım.
4	En az esnekliğe sahip olanlardan biriyim.
5	En az esnekliğe sahip olan benim.
	**Bence…**
1	Çok kiloluyum.
2	Olması gerekenden biraz daha kiloluyum.
3	Kilom normal.
4	Olması gerekenden biraz daha zayıfım.
5	Olması gerekenden çok daha zayıfım.
	**Benim yaşımdaki diğer erkeklerle/kızlarla kıyaslandığımda…**
1	En kilolu olan benim.
2	En kilolu olanlardan biriyim.
3	Ne en fazla kilolu ne de en az kilolu olanım.
4	En az kilolu olanlardan biriyim.
5	En az kilolu olan benim.
